# Performance of Dual-Layer Flat-Panel Detectors

**DOI:** 10.3390/diagnostics15151889

**Published:** 2025-07-28

**Authors:** Dong Sik Kim, Dayeon Lee

**Affiliations:** Division of Semiconductor and Electronics Engineering, Hankuk University of Foreign Studies, Yongin-si 17035, Gyeonggi-do, Republic of Korea; yeooon@hufs.ac.kr

**Keywords:** convex combination image, detective quantum efficiency, dual-layer flat-panel detector (DFD), modulation transfer function, noise power spectrum

## Abstract

**Background/Objectives:** In digital radiography imaging, dual-layer flat-panel detectors (DFDs), in which two flat-panel detector layers are stacked with a minimal distance between the layers and appropriate alignment, are commonly used in material decompositions as dual-energy applications with a single x-ray exposure. DFDs also enable more efficient use of incident photons, resulting in x-ray images with improved noise power spectrum (NPS) and detection quantum efficiency (DQE) performances as single-energy applications. Purpose: Although the development of DFD systems for material decomposition applications is actively underway, there is a lack of research on whether single-energy applications of DFD can achieve better performance than the single-layer case. In this paper, we experimentally observe the DFD performance in terms of the modulation transfer function (MTF), NPS, and DQE with discussions. **Methods:** Using prototypes of DFD, we experimentally measure the MTF, NPS, and DQE of the convex combination of the images acquired from the upper and lower detector layers of DFD. To optimize DFD performance, a two-step image registration is performed, where subpixel registration based on the maximum amplitude response to the transform based on the Fourier shift theorem and an affine transformation using cubic interpolation are adopted. The DFD performance is analyzed and discussed through extensive experiments for various scintillator thicknesses, x-ray beam conditions, and incident doses. **Results:** Under the RQA 9 beam conditions of 2.7 μGy dose, the DFD with the upper and lower scintillator thicknesses of 0.5 mm could achieve a zero-frequency DQE of 75%, compared to 56% when using a single-layer detector. This implies that the DFD using 75 % of the incident dose of a single-layer detector can provide the same signal-to-noise ratio as a single-layer detector. **Conclusions:** In single-energy radiography imaging, DFD can provide better NPS and DQE performances than the case of the single-layer detector, especially at relatively high x-ray energies, which enables low-dose imaging.

## 1. Introduction

In digital radiography imaging, dual-layer detectors, where two detector layers are stacked together, have two main applications: conducting material decomposition and obtaining high performance, enabling spectral imaging with a single exposure. Applications for the material decomposition, such as the dual-energy subtraction (DES) for bone and tissue separations, bone mineral density (BMD) measurement, and metal artifact correction, are based on energy-selective imaging from copper (Cu) filtering or beam hardening [1,2,3,4,5,6,7,8,9]. For the performance improvement applications, the dual-layer detector can improve or increase the contrast-to-noise ratio (CNR) and detective quantum efficiency (DQE) [10,11,12], and can decrease the noise power spectrum (NPS) [11,13,14,15]. The image quality improvement of the dual-layer detector was also verified by observing the IQFInv values using the CDRAD phantom [12]. Furthermore, multi-layer detectors can implement sophisticate material decompositions and super-resolution imaging [16,17,18,19].

Early dual-layer detectors were made by stacking computed radiography (CR) plates [1,2,3,4,5]. The complementary metal-oxide-semiconductor (CMOS) image sensors and various types of scintillators were also used [6]. Recently, the flat-panel detector of digital radiography (DR) has been used to configure a dual-layer flat-panel detector (DFD) to obtain higher resolution and higher quality dual-energy images [11,12,14,19,20,21,22,23].

**Figure 1:**
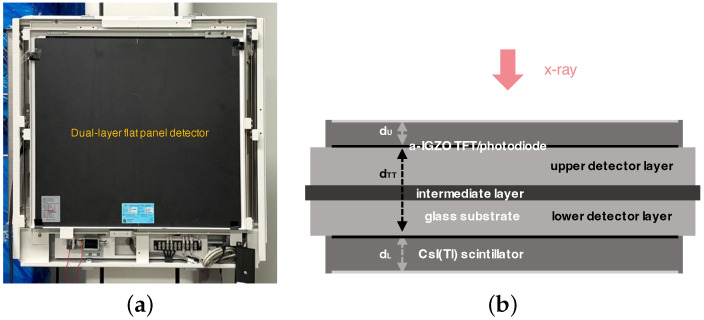
Structure of the dual-layer flat-panel detector (DFD) (DRTECH Co. Ltd., Seongnam, Republic of Korea, www.drtech.com (accessed on 27 July 2025)). The prototype DFD comprises stacked indirect-conversion detectors with CsI(Tl)-scintillator layers, where the pixel pitch is 0.14 mm/pixel and the image size is 3072 × 3072 pixels. (**a**) Appearance of DFD. (**b**) Structure of DFD. The glass substrates of the two detector layers are attached to each other. The lower detector layer is attached opposite to the direction of the incident x-ray [11]. The distance between a-IGZO TFT layers is $d_{\mathrm{TT}}$. The thicknesses of the upper and lower scintillator layers are $d_{U}$ and $d_{L}$, respectively. The pixels on the lower layer may not be in the same position as the pixels on the upper layer.

As illustrated in Figure 1, the structure of DFD consists of two flat-panel detector layers; the upper and lower layers are stacked, and the lower layer usually absorbs relatively high-energy x-ray photons due to the beam hardening or spectrum shaping through filtering [1,9]. The lower layer has a thick scintillator to absorb as many x-ray photons as possible that are not used in the upper layer and that pass through. The thickness of the scintillator of DFD introduced in the literature ranges from 0.2 to 0.6 mm. Various combinations of the detector directions of the upper and lower layers are compared to choose the best combination [11]. In Figure 1 as an example, the lower layer is inverted. Because the inverted layer has a back-irradiation geometry, the modulation transfer function (MTF) performance is better than that of the normal case [24,25]. The intermediate layer between the upper and lower layers can prevent mutual transmission of light photons and can contain a spectral filter such as the Cu filter [1,2,5]. A removable liquid filter composed of iodine (I) can also be used to implement a system that allows selective switching of applications in dual or single-energy imaging [22].

To maintain a uniform registration and magnification between images acquired from the two layers over a wide range of the source-to-image distance (SID), we stack two layers so that the distance between the thin-film-transistor (TFT) layers is as small as possible. Note that the distances in the literature range of 1.1–6.6 mm [23]. However, the closer the two layers are, the more the TFT arrays interfere with each other. Hence, the TFT scan circuit should be designed so that the charges from the detector elements do not interfere with each other when scanning and reading them. Table 1 summarizes various DFD cases introduced in the literature, where the upper and lower layers are mostly composed of indirect-conversion radiography detectors with a scintillator layer of cesium iodide (CsI). However, several cases of the lower layer uses Gd_2_O_2_S (GOS) scintillator layers. The photodiodes are controlled by amorphous silicon (a-Si) [11,14,19,20,21,22] or amorphous InGaZn (a-IGZO) [11,12,23] TFT layers. The pixel pitches are in a range of 0.14–0.15 mm/pixel and the image sizes are in a range of 2880 × 2880–3072 × 3072.

As summarized in Table 2, research was also conducted to construct a triple-layer flat-panel detector by inserting another CsI-scintillator detector instead of using a metal filter in the middle layer [16,17,18].

By using a physical positioning device, the rotational deviation between layers can be controlled to be as small as possible while stacking layers. However, physically aligning the detector-element positions of the lower TFT layer with respect to those of the upper layer is not easy [11]. Hence, the subpixel translocations of the image acquired from the lower layer exist in both horizontal and vertical directions compared to that of the upper layer. Instead of physically aligning the positions of pixels while stacking the layers, we conduct subpixel registration [28] to estimate a subpixel-accuracy translation and then transform the images acquired from the lower layer based on the Fourier shift theorem [29]. Note that the subpixel registration is important in obtaining aligned image pairs as well as inspecting uniform pixel alignment of stacked detector layers [19].

Although the development of DFD systems for material decomposition applications is actively underway, there is a lack of research on whether single-energy applications of DFD can achieve better performance than the single-layer case. In this paper, we introduce several prototypes of DFD (DRTECH Co. Ltd., www.drtech.com) which are currently being developed and conduct extensive experiments to observe their performances in terms of MTF, NPS, and DQE for different combinations of the upper and lower scintillator thicknesses under the RQA 5 and RQA 9 beam conditions. We also observe the DFD performance for different incident doses. We discuss the alignment issue in constructing DFD and also experimentally observe the DFD performance in terms of the DFD alignment. We finally discuss on obtaining low-dose radiography images due to the improved DQE performance. When acquiring radiography images by increasing the x-ray tube voltage, the DQE performance of single-layer detectors usually deteriorate. However, using convex images acquired from DFD, improved DQE can be obtained even at high tube voltages. Therefore, by using DFD, it is possible to acquire radiographic images with stable high DQE performance even at a wide range of x-ray tube voltages.

This paper is organized in the following way. In Section 2, we first introduce the DFD, which is the target of performance evaluation, and briefly introduce the measurement methods of MTF, NPS, and DQE, which are methods for evaluating the performance of the DFD. In Section 3, the experimental results for the DFD evaluation are introduced. In Section 4, discussions on the alignment issue in constructing DFD and obtaining low-dose radiography images are introduced. The conclusion is then stated in the last section.

## 2. Materials and Methods

**Figure 2:**
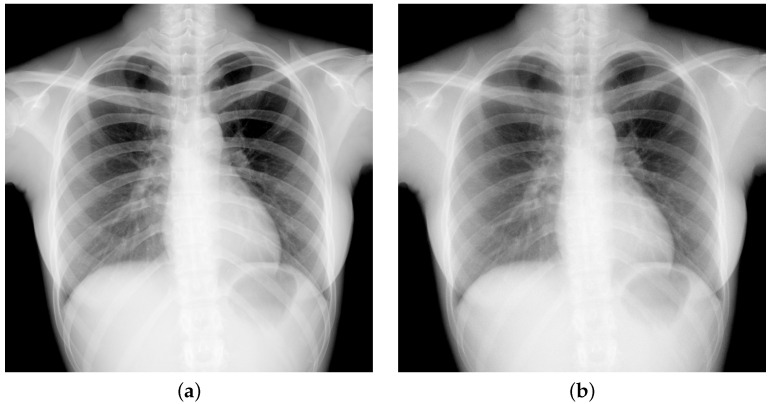
X-ray images acquired from the DFD of $d_{U}=d_{L}=0.5$ mm. The x-ray tube voltage was 120 kVp at an incident dose of 4.028 μGy with 21-mm Al filter. SID was 1800 mm and a 215-lp/inch antiscatter grid was used. (**a**) Upper image acquired from the upper layer (mean: 8270). (**b**) Lower image acquired from the lower layer (mean: 2367).

We consider the DFDs, which are prototypes developed by DRTECH Co. Ltd., South, Korea (DRTECH Co. Ltd., www.drtech.com). As shown in Figure 1b, indirect-conversion flat-panel detectors with CsI(Tl)-scintillator layers are stacked as the upper and lower layers, where the pixel pitch is 0.14 mm and the image size is 3072 × 3072 pixels with a resolution of 16 bits/pixel. For the various thickness combinations for the experiments, we considered the upper scintillator thickness range as $d_{U}$ = 0.2, 0.35, 0.5 mm, and the lower scintillator thickness range as $d_{L}$ = 0.35 and 0.5 mm. The lower layer detector is attached at an inverted direction to the incident x-rays to improve the MTF performance. Here, to minimize the projection difference between the upper and lower images, acquired from the upper and lower layers of DFD, the distance between the TFT panels is as thin as $d_{TT}=1.1$ mm. For each thickness combination, we suppose that the upper and lower layers are accurately aligned based on the subpixel registration [30] and two-step registration [23], where the amplitude and phase distortion due to the interpolation can be minimized. An example of upper and lower chest images acquired from the DFD with $d_{U}$ = 0.5 mm are shown in Figure 2.

Let $p_{D}\left(u\right)$ denote a convex combination [31] of the upper and lower images $p_{U}$ and $p_{L}$ acquired from a DFD at a pixel position of u. The convex image $p_{D}$ is then defined as

(1) $$\begin{array}{c} p_{D}\left(u\right):=\lambda p_{U}\left(u\right)+\left(1-\lambda \right)p_{L}\left(u\right), \end{array}$$

for a combination coefficient of λ such that λ∈[0,1] [11,14]. Kim [11] optimized the combination coefficient to maximize the SNR or DQE of the convex image. Kim [11] and Wang et al. [14] also considered optimal coefficients to minimize the noise in the convex image. Note that, for practical DFDs, the range of optimal coefficients is λ=0.48–0.53 for relatively low frequencies. Simply adding the upper and lower images without any optimization process (λ=0.5), we can also improve the SNR and DQE performance. In this paper, the performance of DFD is observed through the convex image $p_{D}$.

### 2.1. Modulation Transfer Function

The frequency performance of imaging systems can be evaluated by measuring the detector MTF, which is the normalized amplitude response acquired from the Fourier transform of the impulse response or the point spread function. We first measure directional MTF curves from the convex image $p_{D}$ based on the method described in the IEC standard [32]. Here, to avoid overlaps of aliases and increase measurement accuracies [33], the oversampled impulse response is calculated from the derivative of the oversampled step response, which is acquired from upper and lower x-ray images using a tungsten slant-edge phantom with a slant angle of θ. Note that the maximum frequency is $f_{s}/2\cos\left(\theta \right)$ in line pairs per millimeter (lp/mm), where $f_{s}$ is the detector sampling frequency, and the frequency axis of the measured MTF should be compensated by dividing cos(θ). An odd sampling space occurs at every ⌈1/tan(θ)⌉th sample [34]. In order to minimize the influence of this location on the oversampled impulse response, the vertical position of the starting horizontal line for the oversampled step response is selected so that the measured MTF values are maximized.

Let $\mu_{U}$ and $\mu_{L}$ denote the means of the images from the upper and lower layers, respectively. The mean of $p_{D}$ is then given as $\mu_{D}:=\lambda \mu_{U}+\left(1-\lambda \right)\mu_{L}$. The amplitude impulse response of the convex image $p_{D}$ satisfies $\lambda \mu_{U}T_{U}\left(\omega \right)+\left(1-\lambda \right)\mu_{L}T_{L}\left(\omega \right)$, where $T_{U}$ and $T_{L}$ denote the upper and lower MTF curves, respectively. The directional MTF of the convex image from DFD, which is denoted as $T_{D}$, is a normalized amplitude response by $\mu_{D}$ and hence satisfies the following relationship [11]:

(2) $$\begin{array}{c} T_{D}\left(\omega \right):=\frac{\lambda \mu_{U}T_{U}\left(\omega \right)+\left(1-\lambda \right)\mu_{L}T_{L}\left(\omega \right)}{\mu_{D}}. \end{array}$$

In (2), ω is the normalized radian frequency given as $\omega :=2\pi f/f_{s}$. Note that the MTF of the convex image is also a convex combination as (2) and thus is between those of the upper and lower layers. Therefore, the MTF performance of $p_{D}$ cannot be improved in the DFD structure compared to the case of a single layer of upper or lower detector layers. However, as discussed in the follow sections, the NPS and DQE performance of DFD can be improved compared to the single-layer case. On the other hand, if the overall DQE performance of DFD is kept the same as that of a single-layer detector, a thinner scintillator thickness can be used and thus the overall MTF curve can be improved through the DFD.

### 2.2. Noise Power Spectrum

For a given frequency, the normalized periodogram mean of the convex image is greater than or equal to the harmonic mean of those of upper and lower images and asymptotically equal to the normalized NPS (NNPS) of DFD. Let $P_{D}$ denote the directional NNPS of the convex image acquired from DFD. $P_{D}$ can then have an approximate relationship:

(3) $$\begin{array}{c} \min_{\lambda }P_{D}\left(\omega \right)\approx {\left[\frac{1}{P_{U}\left(\omega \right)}+\frac{1}{P_{L}\left(\omega \right)}\right]}^{-1} \end{array}$$

in ${\mathrm{mm}}^{2}$, where $P_{U}$ and $P_{L}$ denote the NNPSs of the upper and lower layers, respectively [11]. We notice that $\min_{\lambda }P_{D}$ is approximately less than both $P_{U}$ and $P_{L}$ due to the harmonic mean relationship of (3). Hence, the NNPS of the convex image can be better than those of the upper and lower layers. If $P_{U}$ is equal to $P_{L}$, then the directional NNPS is minimized as $\min_{\lambda }P_{D}\approx P_{U}/2$.

### 2.3. Detective Quantum Efficiency

For the convex image acquired from DFD, the corresponding DQE denoted as $Q_{D}$ can be derived from $Q_{D}\left(\omega \right):=T_{D}^{2}\left(\omega \right)/q_{0}dP_{D}\left(\omega \right)$, where $q_{0}$ is the mean quanta per area and dose (1/mm^2^Gy), and *d* is the incident dose (μGy) [32]. An optimal coefficient of λ for maximizing $Q_{D}$ can be calculated by using the upper and lower NPS and MTF curves for a fixed ω. For an optimal combination coefficient of λ, we can asymptotically obtain an algebraic DQE summation of

(4) $$\begin{array}{c} \max_{\lambda }Q_{D}\left(\omega \right)\approx Q_{U}\left(\omega \right)+Q_{L}\left(\omega \right) \end{array}$$

for a fixed ω, where $Q_{U}$ and $Q_{L}$ are the upper and lower DQE values, respectively [11]. Hence, we can significantly improve the detector DQE performance from DFD compared to the single-layer detector.

## 3. Results

**Figure 3:**
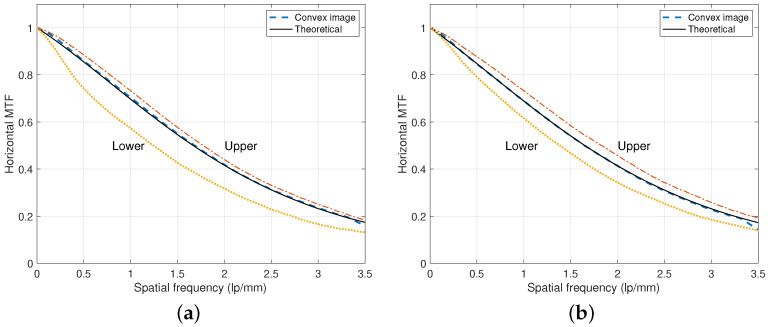
Directional MTF of the DFD in Figure 1. The MTF curves from the convex, upper, and lower images were acquired from a DFD of $d_{U}=0.35$ mm and $d_{L}=0.5$ mm. The MTF from the convex image is between those of the upper and lower detector layers from the convex combination of (1). (**a**) MTF curves under the RQA 5 beam condition with 70 kVp and 12.9 μGy. (**b**) MTF curves under the RQA 9 beam condition with 120 kVp and 12.7 μGy.

In Figure 3, experiments on measuring directional MTF curves are illustrated for the case of $d_{U}=0.35$ mm and $d_{L}=0.5$ mm. Here, the theoretical curves are derived based on a parametric model on MTF [11]. We can observe that the MTF curves of the convex images are very close to the corresponding theoretical curves that are between the upper and lower MTF curves. For the lower x-ray tube voltage case of RQA 5 (70 kVp), the MTF curve of the convex image is very close to that of the upper layer compared to the RQA 9 (120 kVp) case. We notice that the MTF of the lower layer is usually lower than that of the upper layer due to the thicker scintillator. However, as shown in the DFD structure of Figure 1, the inverted lower layer to the x-ray direction has a back-irradiation geometry and can improve the degraded MTF performance of the lower layer [24].

**Figure 4:**
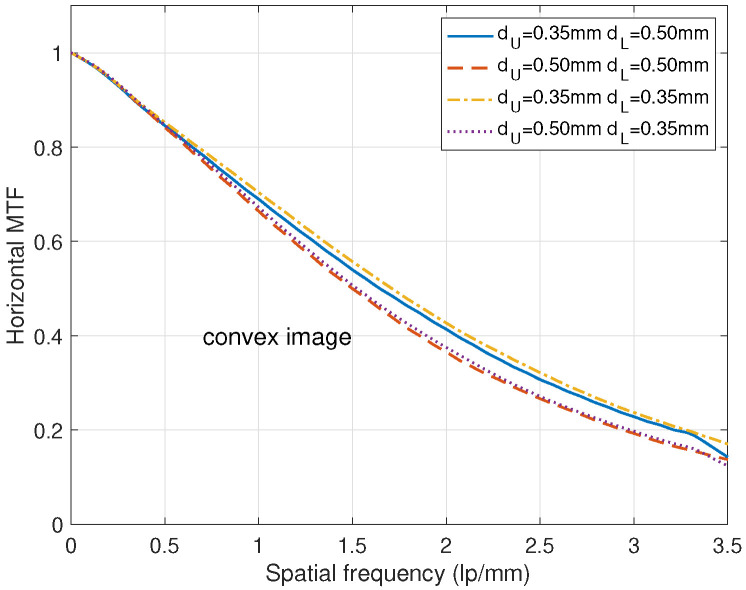
MTF comparison for various combinations of $d_{U}$ and $d_{L}$, where the beam condition was RQA 9.

Figure 4 illustrates the MTF curves for various combinations of the scintillator thicknesses. For a fixed lower thickness $d_{L}=0.35$ mm or $d_{L}=0.5$ mm, the DFD that has a thicker scintillator layer shows a lower MTF curve. For the DFD where $d_{U}=d_{L}=$ 0.5 mm, the overall thickness is the thickest, thus the MTF curve is the lowest. It is clear that the DFD with $d_{U}=d_{L}=0.35$ mm shows the best MTF performance. When taking edge phantom images to obtain MTF characteristics, the dose is generally set high to reduce the influence of x-ray photon noise on the MTF curve.

**Figure 5:**
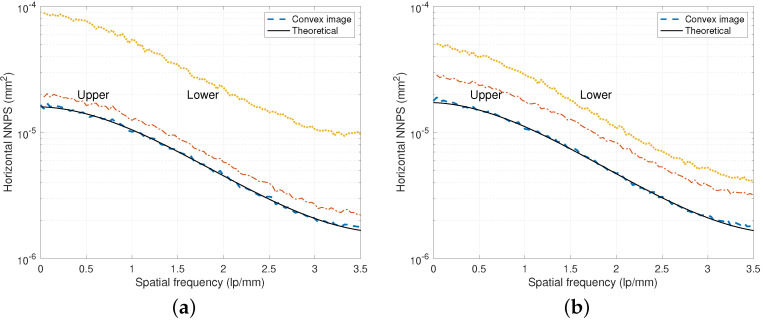
Directional NNPS of the DFD in Figure 1. The NNPS curves from the convex, upper, and lower images were acquired from a DFD of $d_{U}=0.35$ mm and $d_{L}=0.5$ mm. The NNPS of the convex image is smaller than those of the upper and lower detector layers from the harmonic mean of (3). (**a**) NNPS curves under the RQA 5 beam condition with 2.65 μGy. (**b**) MTF curves under the RQA 9 beam condition with 120 kVp and 2.52 μGy.

Figure 5 illustrates measurement examples of the directional NNPS for the DFD prototypes of Figure 1. Here, we compensated the NNPS values, which were inflated during the gain correction procedure, by considering the number of white images acquired under an incident exposure for the gain map design [15,35]. We can observe that the NNPS value of the upper layer is less or better than that of the lower layer, and the convex image NNPS is less than that of the upper layer. Hence, to improve the noise performance, the images from both upper and lower layers should be convex combined as shown in (1) for appropriate combination coefficients of λ. As mentioned after (1), this convex combination coefficient λ can be chosen as 0.5 in most cases [11].

**Figure 6:**
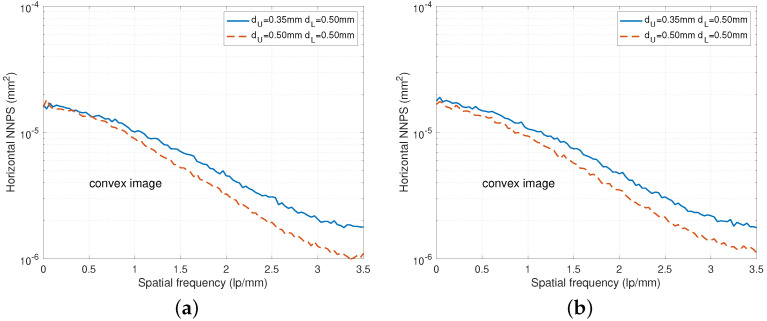
NNPS comparison for various combinations of $d_{U}$ and $d_{L}$. (**a**) NNPS curves under RQA 5. (**b**) NNPS curves under RQA 9.

To further improve the NPS performance, we can increase the upper scintillator thickness as observed in Figure 6 for both RQA 5 and RQA 9. However, increasing this thickness has the side effect of worsening the upper MTF performance and lower NPS performance as shown in Figure 4 and Figure 6, respectively.

**Figure 7:**
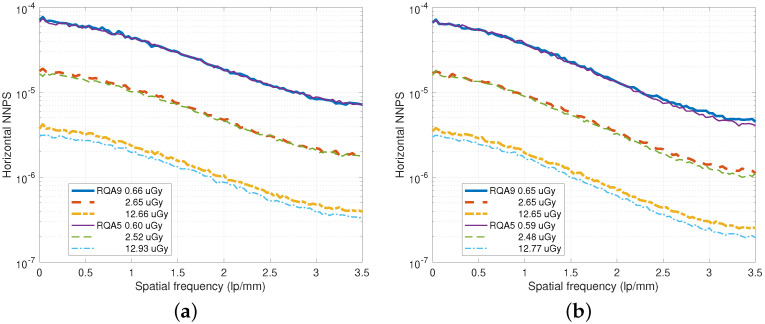
NNPS curves for various incident doses under the RQA 5 and RQA 9 beam conditions. (**a**) NNPS curves for a DFD of $d_{U}=0.35$ mm and $d_{L}=0.5$ mm. (**b**) NNPS curves for a DFD of $d_{U}=d_{L}=0.5$ mm.

In Figure 7, NNPS curves for various incident doses are illustrated. Because the upper thickness of Figure 7b is thicker than the case of Figure 7a, the NNPS performance improves as the spatial frequency increases while the MTF performance degrades as shown in Figure 4.

**Figure 8:**
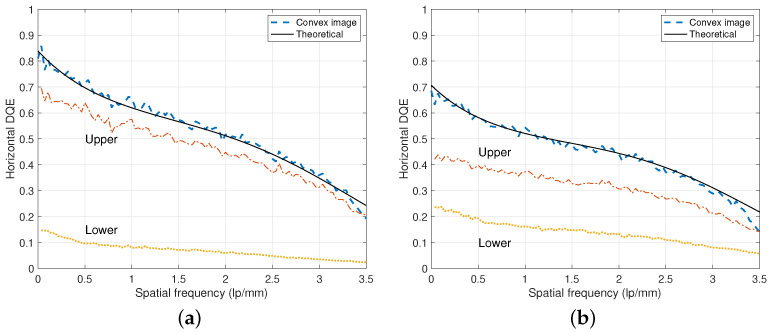
Directional DQE of the DFD in Figure 1. The DQE curves from the convex, upper, and lower images were acquired from a DFD of $d_{U}=0.35$ mm and $d_{L}=0.5$ mm. The DQE of the convex image is the summation of those of the upper and lower layers from the algebraic summation of (4). (**a**) DQE curves under the RQA 5 beam condition with 2.65 μGy. (**b**) DQE curves under the RQA 9 beam condition with 120 kVp and 2.52 μGy.

Under the RQA 5 and RQA 9 beam conditions, DQE experiments for a prototype of DFD with $d_{U}=0.35$ mm and $d_{L}=0.5$ mm are illustrated in Figure 8. In the case of RQA 5 in Figure 8a, since more photons are absorbed in the upper layer, the DQE of the upper image is greater than that of RQA 9 in Figure 8b. In other words, in the case of a single-layer detector that considers only the upper layer, the DQE decreases and worsens as the x-ray tube voltage increases. On the other hand, in the case of the lower layer, when the x-ray tube voltage increases, the absorption of photons in the upper layer decreases, and the passed photons are absorbed in the lower layer, which increases the DQE value increases as observed in Figure 8b [20].

Using the convex images acquired from DFDs can alleviate this DQE degradation problem in the upper layers that represent single-layer detectors. For the experiments on the DFDs of Figure 1, optimal values of λ are approximately constant for all ω. Hence, from (4), an approximation of $Q_{D}\approx Q_{U}+Q_{L}$ holds for all frequencies and this property can be observed for both cases of RQA 5 and RQA 9 in Figure 8. As observed in Figure 8b, even for the RQA 9 beam condition with 120 kVp, the DFD showed improved DQE values closely to the theoretically achievable values, which are derived based on a parametric model.

**Figure 9:**
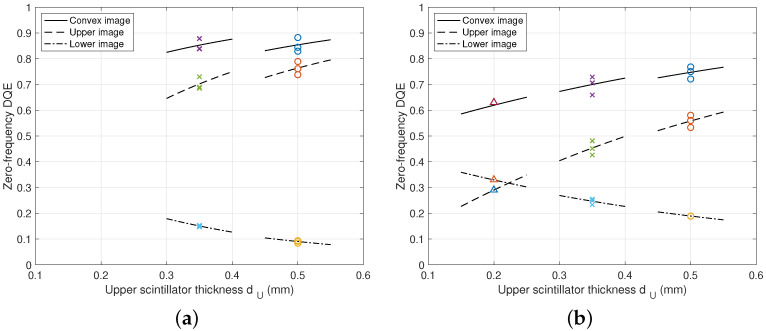
Zero-frequency DQE performance with respect to the upper scintillator thickness $d_{U}$, where $d_{L}=0.5$ mm. Experimental DQE values were obtained for several incident exposures from 0.655 μGy to 12.7 μGy. The same color indicates the results of experiments conducted under the same conditions. (**a**) DQE curves under RQA 5. (**b**) DQE curves under RQA 9.

In Figure 9, the zero-frequency DQE values are plotted with respect to the upper scintillator thickness $d_{U}$, where the lower scintillator thicknesses are fixed as $d_{L}=0.5$ mm. We can observe that as the thickness of the upper layer increases, the upper DQE value increases while the lower DQE value decreases. Furthermore, the convex images of DFD can provide improved DQE values even for the RQA 9 beam condition. As observed in Figure 8a, the upper DQE curve for the RQA 5 beam condition shows a saturated shape and similar values for the $d_{U}=0.35$ mm and $d_{U}=0.5$ mm cases. However, under the RQA 9 beam condition of Figure 8b, increasing $d_{U}$ can obtain more DQE gain, thus it is necessary to select $d_{U}=0.5$ mm rather than the case of $d_{U}=0.35$ mm. For example, the RQA 5 beam condition of Figure 8a shows a DQE of $Q_{U}\left(0\right)=0.689$ and the RQA 9 beam condition of Figure 8b shows a decreased value of $Q_{U}\left(0\right)=0.451$. On the other hand, for the lower layers, $Q_{L}\left(0\right)=0.150$ of RQA 5 increases to $Q_{L}\left(0\right)=0.255$ of RQA 9. The convex image from DFD can provide improved DQE values of $Q_{D}\left(0\right)=0.839$ and $Q_{D}\left(0\right)=0.706$ for the beam conditions of RQA 5 and RQA 9, respectively.

**Figure 10:**
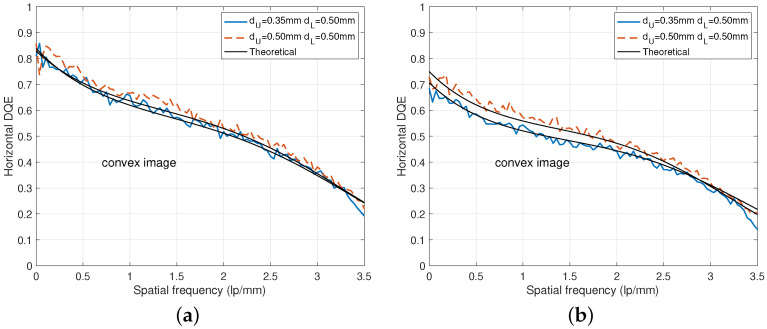
DQE comparison for various combinations of $d_{U}$ and $d_{L}$. (**a**) DQE curves under RQA 5. (**b**) DQE curves under RQA 9.

In Figure 10, examples of DQE curves for different scintillator thicknesses and beam conditions. The DQE curves for the two thickness combinations under the RQA 5 beam condition in Figure 10a show similar values as discussed in Figure 9a. For the RQA 9 beam condition in Figure 10b, it is better to choose the combination of $d_{U}=d_{L}=0.5$ mm because the DQE curve increases to a meaningful level. For example, the RQA 5 beam condition shows similar zero-frequency DQE values of 0.839 and 0.829 for both thickness combinations. On the other hand, for the RQA 9 beam condition, the zero-frequency DQE value of $d_{U}=0.35$ mm is 0.689 and increases to 0.761 for $d_{U}=0.5$ mm.

**Figure 11:**
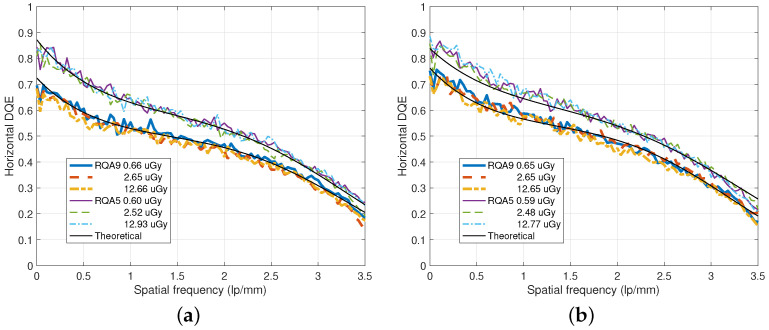
DQE curves for various incident x-ray doses under the RQA 5 and RQA 9 beam conditions. (**a**) DQE curves for a DFD of $d_{U}=0.35$ mm and $d_{L}=0.5$ mm. (**b**) DQE curves for a DFD of $d_{U}=d_{L}=0.5$ mm.

In Figure 11, DQE values for various incident doses are illustrated. We can observe that the DQE performance is insensitive to the incident x-ray dose. As observed in Figure 9, the RQA 5 beam condition yields higher DQE values than the RQA 9 case.

## 4. Discussion

In this section, we introduce further discussions in addition to the performance evaluation and discussion of DFD through experiments in the previous section. We first discuss the alignment issue to maximize DFD performance in terms of MTF, NPS, and DQE. Convex images acquired from DFD can achieve improved DQE, which allows for obtaining x-ray images of the same quality even at lower doses. We next discuss this low-dose image acquisition issue.

### 4.1. Alignment of Lower Images

Current subpixel registration methods generally use entire pixels of natural scenes and thus are not suitable to find such a fine translation of subpixel resolutions for the alignment purpose of DFD [36,37]. Shi et al. [38] used an affine transform based on an interpolation with the IsoCal phantom [39]. Wang et al. [14] also used an interpolation scheme. However, the interpolation-based transforms cause amplitude and phase distortions [30].

Instead of considering a special phantom for subpixel registration, Kim [11] used the conventional slant-edge phantom, which is generally employed in measuring MTF, and maximized the DQE value to obtain an aligned convex combination. Lee and Kim [30] recently conducted a maximum-amplitude subpixel registration based on an amplitude response of MTF, where a cyclic-coordinate optimization is conducted. Note that this method can find local subpixel translations using a small portion of the slant edge. Kim and Lee [23] proposed a two-step image registration method to cope the spatial translation while stacking the layers and the scale due to the x-ray projection in acquiring images from DFD [12]. To align the DFDs of the manuscript, we used this two-step image registration method. The first step is translating the lower image based on the Fourier shift theorem using the estimated translations from the subpixel registration scheme [30]. The second step is conducting scaling based on an affine transform with the scale factor $1+d_{TT}/d_{SID}$, where $d_{SID}$ is the SID value. Here, a cubic interpolation scheme is used.

**Figure 12:**
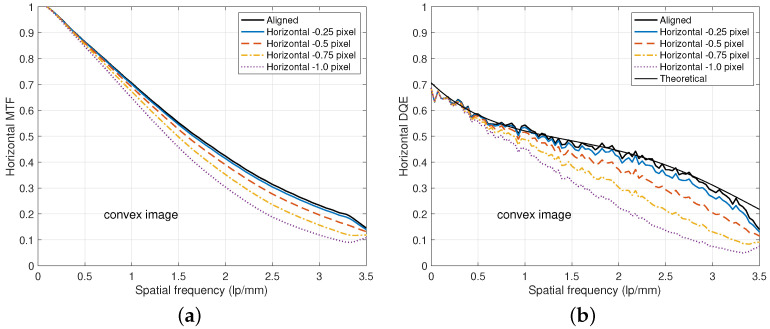
Degradations due to various horizontal alignment errors, where the DFD has $d_{U}=0.35$ mm and $d_{L}=0.5$ mm, and the beam condition is RQA 9. (**a**) MTF degradation. (**b**) DQE degradation.

Figure 12 shows an example of performance degradation due to misaligned DFDs. If the lower image is not aligned, then the MTF values of relatively high frequencies are usually lower than the theoretical ones as illustrated in Figure 12a, where the horizontal misalignment deviations are from −0.25 to −1.0 pixel. Consequently, the DQE values decrease as shown in Figure 12b. In particular, MTF becomes worse than that of the lower layer and DQE becomes worse than that of the upper layer. Hence, precise alignment of the lower detector layer based on an image registration is important to ensure improved MTF and DQE performances even at relatively high frequencies.

**Figure 13:**
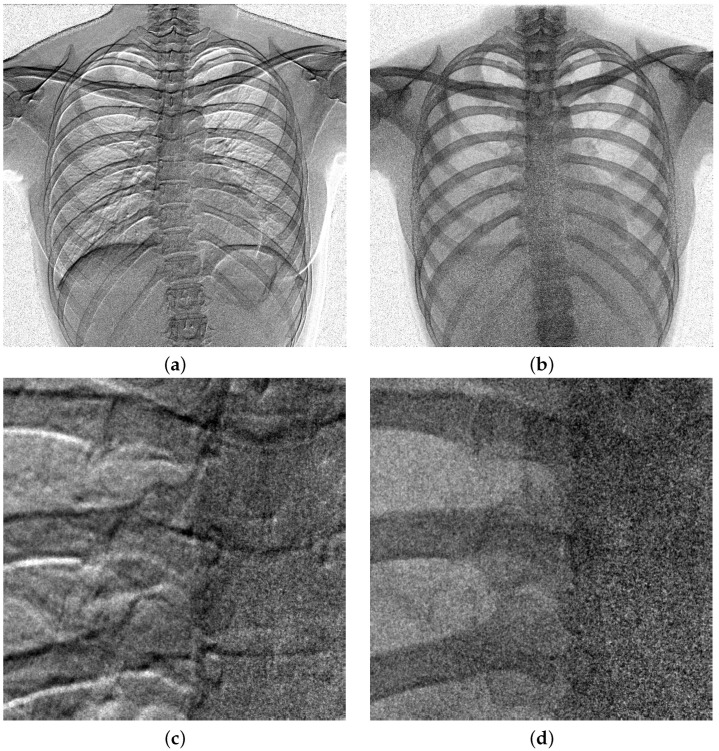
DFD alignment example for the images of Figure 2. The ratio of the upper and lower images is displayed to make it easier to observe the misalignment in detail. (**a**,**c**) Without alignment with a translation error of (−8.946, 2.977), which is estimated from a maximum-amplitude subpixel registration method [30]. (**b**,**d**) Aligned result with the two-step registration method [23].

Figure 13 shows an example of misalignments between the upper and lower images acquired from the DFD. To observe the misalignment, we use the ratio of the upper and lower images. Figure 13b,d show aligned ratio images, while Figure 13a,c show echo shape distortions due to alignment errors.

### 4.2. Low-Dose Radiography Imaging

Improving the DQE performance of the detector is advantageous in obtaining radiography images at low doses. Because the SNR obtained from the detector is given by $T_{D}\left(\omega \right)/\sqrt{P_{D}\left(\omega \right)}=\sqrt{Q_{D}\left(\omega \right)q_{o}d}$, if the DQE value of the detector increases by the ratio *r*, an image with the same SNR can be obtained even if the dose decreases by the inverse of that ratio, the dose reduction 1/r.

**Figure 14:**
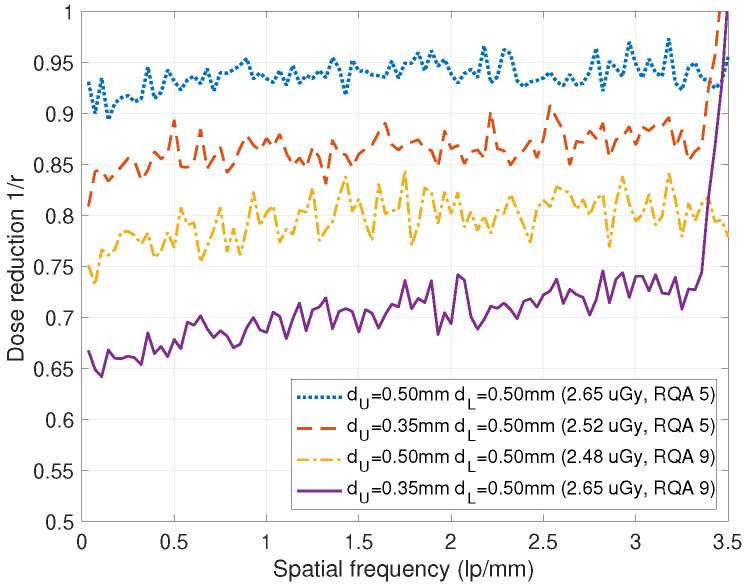
Dose reduction 1/r for the DFDs of Figure 1 for the beam qualities RQA 5 and RQA 9, where *r* is the DQE of DFD divided by the DQE of the upper detector. DFD can provide the same SNR images with reduced incident doses by 1/r.

In Figure 14, the dose reduction 1/r for various combinations of the scintillator thicknesses in the DFDs of Figure 1 are illustrated, where the upper layers are regarded as a conventional single-layer detector. We can observe that the DFD with $d_{U}=0.35$ mm, $d_{L}=0.5$ mm shows the lowest dose reduction of about 0.7. This implies that 70% of the incident dose of a single-layer detector can provide the same SNR for the DFD.

## 5. Conclusions

In this paper, we analyzed the performance of DFD, in which two flat-panel detectors are stacked with a minimal distance between the layers, and the lower image was translated based on the Fourier shift theorem and then scaled based on the cubic interpolation to solve the misalignment problem. The performance of DFD was evaluated by experimentally measuring the MTF, NPS, and DQE of the convex image acquired from DFD. DFD could obtain a better performance than that of the single-layer detector especially at relatively high x-ray tube voltages in terms of both NPS and DQE. Theoretical and experimental observations can be summarized as follows: The MTF of the convex image from DFD is between those of the upper and lower layers. The NNPS of the convex image can be better than those of the upper and lower layers. The DQE of the convex image can be the sum of those of the upper and lower layers.

## Figures and Tables

**Table 1:** Dual-layer flat-panel detectors (DFDs) for single-exposure imaging.

	Intermediate	CsI Scintillator		Distance	
	Filter	$d_{U}$	$d_{L}$	$d_{\mathrm{TT}}$	**Applications**
Lu et al. [20], 2019	1 Cu	0.2	0.55	2.5	D
Shi et al. [21], 2020	1 Cu	0.2	0.55	2.5	D
Mogami et al. [26], 2021	-	CsI	GOS	-	D
Takarabe et al. [27], 2022	-	CsI	GOS	-	P (upper)
Kim [11], 2023	None, 0.5 Cu	0.5	0.5	1.3–2.2	P (convex)
Wang et al. [14], 2023	None	0.55	0.55	-	D, P (convex)
Cai et al. [22], 2023	Liquid 0.76 I	0.2	0.55	-	D, P
Su et al. [19], 2024	1 Cu	0.26	0.55	6.6	S
Kim et al. [12,23], 2025	None	0.35–0.5	0.5	1.1	P (convex)

CsI: cesium iodine (Tl), GOS: ${\mathrm{Gd}}_{2}O_{2}$S (Tb); D: material decomposition, P: performance, S: super-resolution; The unit of thickness is millimeter (mm).

**Table 2:** Triple-layer flat-panel detectors for single-exposure imaging.

	Intermediate	Thickness			
	Filter	$d_{U}$	$d_{M}$	$d_{L}$	**Applications**
Maurino et al. [16], 2016	None	0.05–0.2	0.1–0.7	0.5	D
Jiang et al. [17,18], 2024	None	0.2	0.4	0.55	D

D: material decomposition; The unit of thickness is millimeter (mm).

## Data Availability

The original contributions presented in the study are included in the article, further inquiries can be directed to the corresponding author.

## Abbreviations

DFD	Dual-layer flat-panel detector
DQE	Detective quantum efficiency
MTF	Modulation transfer function
NNPS	Normalized noise power spectrum
NPS	Noise power spectrum
TFT	Thin-film-transistor
SNR	Singal-to-noise ratio
SID	Source-to-image distance
